# The British Medical Association

**Published:** 1905-08-05

**Authors:** 


The Hospital.

A Journal of

The Medical Sciences and Hospital Administration.

Vol. XXXVIII.—No. 984. SATURDAY, AUGUST 5, 1905.

### The British Medical Association.

The non-medical Press of the last few days has given a good deal of prominence to the opinions concerning national physical deterioration which have been expressed during the meeting at Leicester of the British Medical Association, or have been subsequently called forth in the form of comment. We cannot flatter ourselves that these deliverances, so far as they have fallen under our notice, have been calculated to increase the reputation of the profession for scientific caution, or even for the homely virtue of ascertaining facts before speaking with a semblance of authority. They have, for the most part, been characterised by a curious blending of the obviously commonplace with what is called by schoolboys the cocksure. According to one of our evening contemporaries, a "well-known doctor" spoke to a "Press representative" in "a few curt and emphatic words," words which unfortunately seem to have been prompted by an old number of *Punch* which may possibly have adorned his waiting-room. "Environment has great influence," he said, "but heredity is still more powerful." In glancing over a good many reports of the collective wisdom of the meeting, as applied to one of the gravest of national problems, we find very little that is better than this. The public is surely entitled to ask the "well-known doctor" by what tests he would distinguish the influence of heredity from that of environment, and how he would recognise any particular character as being produced by one or by the other? Everybody knows a score of families in comfortable circumstances, families in which the parents are just average people of their class, of fair physique and fair intellectual endowment, but in which the children present great differences of appearance, of development, of character, of capacity, or of all four. In such cases, where the environment has been the same, the differences are commonly attributed to heredity, to "taking after" one parent more than after the other, or where there is marked difference from both parents, to atavism, to inheritance from some known or conjectured earlier ancestor. This may be very good conjecture, and it is occasionally strongly supported by reference to ancestral portraits or histories; but it is only conjecture, and affords no basis for scientific conclusion or action. Sometimes, there is reason to believe, a dormant hereditary influence may be brought into activity by environment. An eminent Scotch physician has placed on record that, after a great commercial failure in Scotland, by which many families were reduced from affluence to poverty, children bearing evidence of inherited syphilis were born to parents who were among the sufferers, and whose earlier children had shown no indications of the taint, although the fathers were known to have contracted the disease, and were supposed to have been cured before marriage. Assuming the facts to be as stated, do they point to heredity or to environment? Did a lowered environment occasion the development, in the younger children, of a taint common to all, but repressed in the elder ones; or did it bring into fresh activity, in the fathers, a disease from which, in their prosperity, the earlier children had escaped? It is easy to argue on either side of the question, but men of science do not argue, they reason. It is only by virtue of reasoning that they can establish a claim to be accepted as guides to public conduct and opinion.

Another melancholy example appears to have been furnished by Dr. Langley Browne, who availed himself of a discussion on physical deterioration to pose as a supporter of a *quasi* papal doctrine of *non possumus* in relation to fiscal arrangements. He assumed, what has from the first been absolutely denied by the advocates of fiscal reform, and has never been established by their opponents, that it would increase the cost of what he indefinitely calls "food"; and on this ground, which he is reported to have described as a "reason," he declared his objection to proposals which have not yet been formulated, and which, at any rate, were not before the meeting. He assumed, moreover, that, even if a working man were earning better wages, he would not give his wife more money with which to meet the "increased cost," thereby evincing a very remarkable want of acquaintance with the ordinary position of the working man's wife in this country, and of her control over the family exchequer. There are, of course, exceptions; but, as a rule, the wife of the decent working man is the business manager of the household, and her errors of expenditure arise from ignorance and not from being stinted by her husband. The other speakers on the occasion do not appear either to have contributed any novelty to the discussion, or even to have thrown any new light upon either the causes or the relative importance of universally recognised factors in the problem. "City life," "overcrowding," and "alcoholism," were trotted out in the usual manner; but these are effects or symptoms rather than actual causes of the evils of which we all complain. The fullest recognition of them leaves untouched the questions which would demand the attention of statesmen, if the genus had not been choked out of existence by the antagonistic development of politicians. They do not tell us why the agricultural population have crowded into towns, nor by what means the habit of alcoholic excess has been made to take root among our people. Still less do they tell us by what means these causes of degradation and misery may be diminished in the future. They dwell upon the obvious and they leave the essential alone. The medical trumpet gives but an uncertain sound, and it is difficult for would-be reformers to make themselves ready for battle.

